# Dr. Bergeon’s Method of Treating Consumption by Means of Gaseous Enemata

**Published:** 1887-05

**Authors:** Byron H. Daggett

**Affiliations:** 258 Franklin street; Buffalo, N. Y.


### *DR. BERGEON'S METHOD OF TREATING CONSUMPTION BY MEANS OF GASEOUS ENEMATA.**

By Dr. BYRON H. DAGGETT, Buffalo, N. Y.

Being urged to carry out the treatment of pulmonary consumption as inaugurated by Prof. Bergeon of France, and not having the apparatus for so doing, I attempted from a meagre description of the proposed method to extemporize an apparatus for fulfilling its indications. Messrs. Howell & Son kindly charged some of their so-called soda water bottles with carbonic dioxide. This carbonic gas bottle was connected by a rubber tube with a Wolff bottle containing nearly a quart of water saturated with hydrogen sulphide, and the Wolff bottle was again connected with a rubber bag having a stop-cock. Pressing upon the lever of the carbonic gas bottle, the gas is forced through the sulphurous water, carrying with it the sulphuretted hydrogen into the bag, and when the bag is filled the stop-cock is turned and a rectal nozzle is introduced into the disconnected tube. The remedy is then ready for use, and is readily carried to the bedside of the patient. An objection made to this manner of generating and using the gas is that too much hydrogen sulphide may be used. My limited experience does not corroborate this view. I have found no ill effects from it, but, upon the other hand, my patients have soon noticed when the sulphurous water was depleted so as not to furnish the full complement of sulphurous gas, and would say, "It is not as strong as before and

*Read at the annual meeting of the Alumni Association, Medical Department, Niagara University, April 12, 1887.

does not do as much good." Another objection has been made, that the bags would soon be destroyed. So far, I have not had any such trouble. This modification of the process certainly renders the treatment of patients much more convenient.

In 1882, Dr. Bergeon inaugurated the treatment of pulmonary consumption by gaseous enemata of carbon dioxide and hydrogen sulphide combined, and in July, 1886, reported wonderful results, so that this subject is attracting wide-spread attention.

Bernard demonstrated, in 1857, that toxic and medicinal agents introduced into the intestinal canal were taken up by the venous system, carried through the portal system, the liver, the hepatic veins and the lungs, and were eliminated by the liver and carried off by the bile, or, if volatile, were exhaled by the lungs. In these ways they were prevented from entering the arterial circulation.

He also demonstrated that hydrogen sulphide could be safely injected into the veins and rectum of a dog. Dr. Bergeon, experimenting upon animals, found that injecting preparations of chlorine, ammonia, ether, turpentine and bromine into the rectum caused violent inflammation.

He introduced a mixture of carbon dioxide and hydrogen sulphide, and learned that this combination was well borne, if free from atmospheric air. Reasoning from these premises and knowing that sulphur is an antiseptic as well as a microcide, he instituted the treatment of pulmonary maladies by gaseous enemata. He reported over two hundred cases treated in this way, and some of them he considers cured.

Dr. Cohen, of Philadelphia, says: "All reported cases show rapid amelioration of all suppurative phenomena; marked diminution of cough, expectoration, dyspnœa and night sweats, being noted within two or three days."

In some of his own cases, there was a similar prompt improvement, but not in all, although they have all done well with one exception; some were very pronounced cases, and there was no hope in the more familiar methods of treatment. Dr. Bruen, after several weeks' treatment of cases in the various stages of the disease, summarizes as follows:

"In nearly all cases, lasting effects have been secured in the reduction of temperature, suspension of night sweats, lessened cough and expectoration, and in some *all physical signs of bronchial catarrh are abolished.*

"The amount of gas introduced has been from three quarts to a gallon at each injection, taking from fifteen to thirty minutes.

"Injections were given twice daily in most cases.

"No injurious effects were observed.

"Effects were negative in only one case.

"The ultimate value of the treatment can only be established by time.

"The mode of action would seem to be antiseptic, and, by reducing suppuration, consequent relief of serious symptoms, permitting the patient to gain by food, exercise and general treatment.

"Thus far the gas appears to be a valuable therapeutic agent, rather than a curative plan of treatment."

In this, Bruen appears to differ with Bergeon, who, after four years' experience, reports specifically, and in detail, individual cases who are well and who present no symptoms of the disease, and whom the doctor regards as cured.

My experience in cases in the past ten days coincides with these reports in this: there is marked amelioration of all the symptoms. In the first case, Mr. W., a severe diarrhœa ceased after beginning this treatment. Pulse rate was reduced from 140 to 90 in three days, respirations from 40 to 24. The sputa became thinner and colored yellowish. There was marked relief to the pain and constringency of the chest. One case reports a cold sweat, followed by a severe chill, the third night after beginning the treatment. With this exception, there has been no night sweats since the second night. My patients lose the hectic flush and look pale. The chill referred to I attributed to the treatment, in part at least, as I had pushed the remedy beyond the regulation amount. In administering the gas, I have noticed that there will be a complete stasis for three or four minutes, and suddenly the bag will rapidly subside with a gurgling noise; this brings on colic, and the gas should be turned off until the distension of the abdomen subsides, which will be three or four minutes more. I have administered from three to six quarts once and twice per day, as I found it convenient. My patients send for the bags after three or four treatments, and administer it themselves, at their own pleasure. My experience is too brief and limited to warrant me in formulating deductions of any results except temporary relief.

This method of Bergeon opens a vast field for investigation, and another avenue for reaching and, we trust, successfully combatting lung as well as other visceral maladies.

258 Franklin street.

